# Eating Disorders Spectrum During the COVID Pandemic: A Systematic Review

**DOI:** 10.3389/fpsyg.2021.663376

**Published:** 2021-09-29

**Authors:** Mario Miniati, Francesca Marzetti, Laura Palagini, Donatella Marazziti, Graziella Orrù, Ciro Conversano, Angelo Gemignani

**Affiliations:** ^1^Department of Clinical and Experimental Medicine, University of Pisa, Pisa, Italy; ^2^Department of Surgical, Medical and Molecular Pathology, Critical and Care Medicine, University of Pisa, Pisa, Italy

**Keywords:** SARS-COV2 disease, COVID-19, pandemic, eating disorders spectrum, bulimia nervosa, anorexia nervosa, binge eating disorder

## Abstract

**Background:** Several data suggest that coronavirus disease 2019 (COVID-19) pandemic may exacerbate or trigger eating disorders (EDs). The aim of this paper was to summarize current literature studies on COVID pandemic and EDs.

**Methods:** Literature search, study selection, methods, and quality evaluation were performed according to the preferred reporting items for systematic review and meta-analyses (PRISMA) guidelines.

**Results:** A systematic search allowed the initial selection of 172 papers; 21 (12.2%) papers were eligible and included in the review. In selected studies, a total number of 29,108 subjects were enrolled (range: 10–11,391; mean/SD: 1,386 ± 2,800), 6,216 were men (21.4%), 22,703 were women (77.9%), and 189 (0.7%) were gender fluid or not declared. The mean age/SD of subjects was 30.2 ± 7.7. About 12 studies (57.1%) were online surveys, 4 (19.0%) were retrospective studies, 2 (9.5%) were qualitative studies, 2 (9.5%) were longitudinal cohort studies, and 1 was a social media survey (4.8%). Their analysis revealed five main findings: (1) changes in the routines of physical activities were related to the worsening of preoccupation on weight/body shape; (2) food access limitation during pandemic represented the risk factors for both triggering and exacerbating EDs; (3) restriction in healthcare facilities contributed to increase anxiety levels and to modify treatment compliance; (4) social isolation was related to the exacerbation of symptoms in patients with EDs who are home-confined with family members; and (5) conflicts and difficulties in relationships with “no way out” were the maintenance factors for ED symptoms, especially in adolescents and young adults.

**Conclusion:** The COVID-19 pandemic had a negative impact on EDs that might be triggered by the exceptional conditions derived from COVID-19-related stress in predisposed subjects. Patients who were already affected by EDs experienced the worsening of their clinical conditions and related quality of life (QoL).

## Research Highlights

- During the COVID-19 pandemic, it is essential that patients with EDs remain in touch with their professional supports, to ensure that their health status is monitored. There is an urgent need to better understand the efficacy of the online delivery of treatments for EDs and to improve both the accessibility and acceptability (Barney et al., 2020).
- We noticed some criticisms of online surveys and long-distance management of patients with EDs, such as limited accessibility, mainly because of technical issues (software, technical support, etc.). Difficulties experienced by clinicians in conducting online visits, the need for improvement in online software for the check of vital signs, and privacy issues are the main limitations of this long-distance approach. Even with these limitations, telemedicine was the easiest way in the midterm for the management of patients with EDs during pandemic.
- We suggest that a better understanding of the COVID-19 consequences on the dietary habits of the general population and patients with EDs needs to be related to a more general assessment of the chronobiological rhythms disruption due to lockdown.
- Long-term consequences of current pandemic need to be explored with follow-up studies and with the adoption of shared outcomes measures, which put an end to the Babel of instruments used.

## Introduction

Changes in everyday activitiesand in lifestyle due to severe acute respiratory syndrome CoV2 (SARS-COV2) [coronavirus disease 2019 (COVID-19)] pandemic and related quarantine measures strongly affected the mental health of the general population (Marazziti et al., 2020; Poli et al., 2020), and of special populations (Conversano et al., 2020; Orrù et al., 2021). These changes may represent an additional burden both for individuals with a pre-existing eating disorders (EDs) and for subjects from general population who are belonging to the so-called “*anorexia-bulimia spectrum*” (Miniati and Marazziti, 2019). A series of factors may have a detrimental impact on psychological well-being, eating habits, and the onset or recovery of EDs, including: disruption to living situations, social distancing restrictions, difficult access to healthcare, and societal changes to food behaviors and technology usage (Ammar et al., 2020). Psychosocial stressors stemming from the COVID-19 pandemic and the resulting stay-at-home confinement may exacerbate ED-related triggers and represent a challenging environment for individuals with anorexia nervosa (AN), bulimia nervosa (BN), and binge eating disorder (BED) (Hensley., 2020; McMenemy., 2020). The hypertrophic sense of control of patients with AN and the intense polarization on body weight and shape may worsen, especially when self-control is altered by the impact of exceptional external factors, such as those occurring during the COVID pandemic and lockdown (Schlegl et al., 2020a,b). Patients with AN and BN may foster skipping meals and restricting calories or, conversely, may increase binge eating due to the availability of food at home brought about by food insecurity and hoarding (Touyz et al., 2020; Weissman et al., 2020). Moreover, preliminary observations focused on the risk of developing EDs in patients who contracted COVID-19, renewing questions on the role of immunologic and neurobiological factors as potential triggers for AN and BN through inflammatory processes, together with behavioral changes induced by the infection, such as loss of appetite and decrease in food intake. A vulnerable population of subjects, such as EDs patients, is at high risk of acute and long-term consequences from COVID-19 pandemic. The relevant restrictions in psychiatric and psychological services and the access limitations both to inpatients and outpatients facilities need to be also considered.

The aim of this paper is to summarize and comment on current literature studies on the impact of the COVID pandemic on ED spectrum.

## Materials and Methods

We conducted a systematic search of a few literature studies on online databases, including the studies published between January 1, 2020 and October 31, 2020 using PubMed. We adhered to the preferred reporting items for systematic review and meta-analyses (PRISMA) guidelines in the completion of a systematic review (Moher et al., 2009).

### Protocol and Registration

This review is not included in a research protocol.

### Eligibility Criteria

The systematic review field of search was determined using the patient, intervention, comparison, and outcome (PICO) strategy (Schardt et al., 2007), as detailed in Table 1. Papers were selected for a full-text analysis based on the title, abstract, and keywords, provided that they met the following criteria: (1) written in English; (2) original articles on studies with a longitudinal design; (3) prospective or retrospective, observational (analytical or descriptive), experimental or quasi-experimental, and controlled or non-controlled studies; and (4) the articles accepted for publication in a peer-reviewed journal. Reviews and non-original articles (i.e., case reports, editorials, letters to the editor, and book chapters) were not included. We found no randomized controlled trials (RCTs) or long-term follow-up studies derived from RCTs, therefore, the assessment of the risk of bias of individual studies was not performed.

**Table 1:** Eligibility criteria.

**Systematic review components**	**Inclusion criteria**	**Exclusion criteria**
Population	EDs subjects (Anorexia, Bulimia, Binge Eating), during COVID-19 outbreak and lockdown of each country.	Subjects with altered eating due to the direct effect of COVID infection
Intervention	Online interventions (i.e., use of electronic, digital, or mobile devices to support subjects), or face-to-face interventions	Any type of intervention not focused on EDs
Comparison	Face-to-face interventions, online interventions, subjects with AN, or BN, or BED	Not applicable
Outcomes	Primary outcome: prevalence of EDs during pandemic Secondary outcomes: effects of pandemic on subjects’ outcomes (e.g., patients’ modification of daily routines, dietary habits, physical exercise, QoL).	Not applicable
Study design	Original articles on studies with a longitudinal design; prospective or retrospective, observational (analytical or descriptive), experimental or quasi-experimental, controlled or non-controlled studies; articles accepted for publication in a peer-reviewed journal, written in English.	Reviews and non-original articles (i.e., case reports, editorials, letters to the Editor and book chapters) Paper published not in English

### Information Source and Strategy

A search of literature studies was designed and performed independently in duplicate by two authors (MM and FM).

The MEDLINE search was conducted using the following syntax: [‘feeding and eating disorders’[MeSH Terms] OR (‘feeding’[All Fields] AND ‘eating’[All Fields] AND ‘disorders’[All Fields]) OR ‘feeding and eating disorders’[All Fields] OR (‘eating’[All Fields] AND ‘disorders’[All Fields]) OR ‘eating disorders’[All Fields] OR (‘bulimia’[MeSH Terms] OR ‘bulimia’[All Fields] OR (‘binge’[All Fields] AND ‘eating’[All Fields]) OR ‘binge eating’[All Fields]) OR (‘bulimia’[MeSH Terms] OR ‘bulimia’[All Fields]) OR (‘anorexia’[MeSH Terms] OR ‘anorexia’[All Fields] OR ‘anorexias’[All Fields]) AND (‘sars cov 2’[MeSH Terms] OR ‘sars cov 2’[All Fields] OR ‘covid’[All Fields] OR ‘covid 19’[MeSH Terms] OR ‘covid 19’[All Fields] OR ‘lockdown’[All Fields])], leading to a total of 172 papers.

### Study Selection

Two authors (MM and FM) independently screened the resulting articles for their methodology and appropriateness for inclusion. A consensus discussion was used to resolve the disagreements of reviewers.

### Data Collection Process and Data Items

Two independent authors (MM and FM) assessed the language suitability and subject matter of each paper and extracted the data reported in Table 2. With respect to the setup of patients, the following factors were obtained from the studies: the number of patients enrolled, age of patients, gender distribution, and diagnostic distribution. With respect to intervention, we collected the information on the eventual treatment administered when available. With respect to outcomes, we extracted the prevalence of EDs, the description of the impact of lockdown and quarantine, and the instruments (i.e., rating scales, questionnaires, etc.).

**Table 2:** Studies on eating disorders spectrum during COVID-19 pandemic.

**Author**	**Design**	**Participants**	**Country**	**Assessment**	**Instruments**	**Main finding**
1. Al-Musharaf, 2020	Online survey	*N* = 638 female students/graduate participants at King Saud University (mean age= 22.0 ± 1.9)	Saudi Arabia	May 2020	EES, PSS-10, GAD-7, PHQ-9, PSQI, GPAQ	Low EE in 52.5% of women; moderate EE in 31.7%, 15.8% high EE in 15.8%. More than 42% were depressed, 27.0% were anxious; 71.0% reported moderate stress and 12.5% severe stress. Fat intake (β = 0.192, *p* = 0.004), number of meals (β = 0.187, *p* < 0.001), sugary food consumption (β = 0.150, *p* < 0.001), BMI (β = 0.149, *p* < 0.001), PSS (β = 0.143, *p* = 0.004), energy intake (β = 0.134, *p* = 0.043), and fast food intake (β = 0.127, *p* = 0.005) positively predicted EES score; increase in family income negatively associated with EES scores.
2. Ammar et al., 2020	Online survey	*N* = 1,047	International	April 2020	SWEMWBS, SMFQ, SLSQL, SSPQL, PSQI, STBQL IPAQ-SF, SDBQL	Home confinement had negative effects on all levels of physical activity, namely: reduction of number of min/day of PA (Pre vs. Post: 108.0 ± 114.2 vs. 71.8 ± 88.2, *p* < 0.001) and day/week (Pre vs. Post: 5.04 ± 2.51 vs. 3.83 ± 2.82, *p* < 0.001); increase of sitting h/day (Pre vs. Post: 5.31 ± 3.65 vs. 8.41 ± 5.11, *p* < 0.001). Unhealthy patterns of food consumption (unhealthy food, eating out of control, snacking between meals, and number of main meals) were reported (Pre vs. Post, *t* = −10.66, *p* < 0.001, *d* = 0.50).
3. Baenas et al., 2020	Online survey	*N* = 74 (mean age: 32.12 ± 12.8)	Spain	Patients enrolled from 14^th^ March to 11^th^ May, 2020.	EDI-2,YFAS-2, SCL-90-R, TCI-R, Semi Structured brief telephone survey during COVID-19 confinement	Level of deterioration in functioning assessed during lockdown, in a sample of patients in treatment before the COVID-19 outbreak. Nineteen patients with ED symptoms and anxiety/depression symptoms had a worsening, especially when low self-directedness was present. Patients increased in weight when adopting less adaptive coping strategies to deal with lockdown. However, 55 patients did not experience a significant worsening of their symptoms. They assessed 32 different dimensions or domains, belonging to EDI-2, YFAS-2, SCL-90-r, and TCI-R but they found a significant difference only on the abovementioned TCI-R domain, as confirmed with a path analysis.
4. Barrea et al., 2020	Retrospective study	*N* = 121 (mean age = 44.9 ± 13.3)	Italy	T0: January–March 2020T1: May 2020 (40 days after lockdown)	PSQI	Quarantine associated with significant reduction in physical activity (Pre vs. Post, 62.5 vs. 39.3%, *p* = 0.004), increase in BMI (Pre vs. Post, 32.6 ± 6.0 vs. 33.3 ± 6.2, *p* < 0.001), decrease in sleep quality (Pre vs. Post PSQI score,: 6.37 ± 3.96 vs. 8.64 ± 3.73, *p* < 0.001). Smart working associated to deterioration of sleep quality, more pronounced in male (M vs. F, ΔPSQI = 151.41 ± 94.33 vs. 87.29 ± 115.52, *p* < 0.001).
5. Branley-Bell and Talbot, 2020	Mixed-methods online survey	*N* = 129 (mean age = 29.3 ± 9.0, 62% current ED, 38% in recovery)	UK	April 2020 (14 days after lockdown)	SWEMWBS, PSS-4, ESSI, SCI, RRS-ED	Participants in remission showed higher mental well-being (17.66 ± 2.48 vs. 16.35 ± 3.24, *p* < 0.05), lower perceived stress (13.10 ± 2.71 vs. 14.66 ± 2.61, *p* < 0.001), higher social support (21.94 ± 6.47 vs. 19.76 ± 5.71, *p* < 0.05) and higher perceived control (62.71 ± 12.74 vs. 54.52 ± 13.15, *p* < 0.001).
6. Brown et al., 2020	Qualitative study	*N* = 10 participants with ED (mean age = 29.6)	UK	May–June 2020 (45 day after lockdown)	Interviews	Lockdown as catalyst for either disordered eating behaviors or the effort to recover, depending on participants’ living and work situation, and ED progression. Structures of social and functional restrictions and accessibility to professional support are crucial determinants for mental well-being.
7. Castellini et al., 2020	Longitudinal cohort study	*N* = 169 (44% patients with AN or BN, mean age 31.74 ± 12.76, 56% HC, mean age 30.45 ± 10.89)	Italy	T0: beginning of treatment, January–September 2019 T1: before lockdown, November 2019–January 2020 T2: during lockdown, April–May 2020	BSI, EDE-Q, CTQ-SF, ECR-R, IES-R	T0–T1: improvement in psychopathology (Cohen’s *d* for AN: 0.39; Cohen’s *d* for BN: 0.52) and reduction of monthly objective binge eating (Cohen’s *d* for AN: 0.41; Cohen’s *d* for BN: 1.06). Increase in BMI for AN (Cohen’s *d*: 0.54). T1–T2: increase in compulsive exercise (Cohen’s *d* for AN: 0.32; Cohen’s *d* for BN: 0.30), increase of monthly objective binge eating for BN (Cohen’s *d*: 0.32). AN reported an increase in BMI (Cohen’s *d*: 0.71) and specific ED psychopathology (Cohen’s *d*: 0.26). EDs group reported higher COVID-19 related post-traumatic stress symptoms as compared to HC group (22.07 ± 15.90 vs.17.96 ± 11.41, Cohen’s *d*: 0.30). Household arguments associated with a higher increase in pathological physical exercise during lockdown (T1:0.53 ± 1.34, T2:2.33 ± 5.76 vs. T1:0.94 ± 1.61, T2:7.56 ± 11.34, *p* = 0.014, Cohen’s *d* for patients reporting this factor: 0.62). Fear for loved ones safety predicted an increase in binge eating episodes (T1: 1.39 ± 2.13, T2: 2.24 ± 5.17 vs.T1: 0.83 ± 1.47, T2: 5.05 ± 6.99, *p* = 0.012, Cohen’s *d* for patients reporting this factor: 0.67). Risk factors for Covid-19-related PTS symptoms in AN: childhood trauma (β = 0.34, *p* = 0.031), and insecure attachment style (β = 0.38, *p* = 0.044).
8. Clark Bryan et al., 2020	Qualitative study	*N* = 21 patients with AN (mean age = 25.5 ± 5.6) *N* = 28 carers (mean age = 54.0 ± 7.3)	UK	April 2020	Semi-structured interviews	Key themes for AN: reduced access to ED services; disruption to routine and activities in the community; heightened psychological distress and ED symptoms; increased attempts at self-management in recovery Key themes for carers: concern over provision of professional support for patients; increased practical demands placed on careers in lockdown; managing new challenges around patients’ well-being; new opportunities, perspectives, and approaches.
9. Elmacıoğlu et al., 2021	Retrospective cohort study	*N* = 1,036 subjects (mean age 33.0 ± 12.9)	Turkey	From May 6^th^, to May 26^th^, 2020	BMI, age, gender, and the TFEQ-R18	Increase in emotional eating and uncontrolled eating behaviors, in normal and overweight individuals. Body weight had an increase in 35% of the sample. In the process of social isolation, womens’ uncontrolled eating and emotional eating were higher than in men. Only in 151 subjects there were no significant changes in diet.
10. Fernández-Aranda et al., 2020	Retrospective study	*N* = 121 (mean age 33.7 ± 15.8, 72% ED, 28% patients with obesity)	Spain	June–July 2020	CIES	AN group: significant decrease of scores for eating related symptoms (Pre vs. Post, 11.87 ± 6.79 vs. 9.40 ± 5.61, *p* = 0.015), effects of confinement on eating related style (Pre vs. Post, 8.76 ± 9.61 vs. 6.11 ± 6.94, *p* = 0.023) and changes in emotion regulation (Pre vs. Post, 9.47 ± 4.63 vs. 8.33 ± 4.86, *p* = 0.046). Patients with obesity had a significant decrease in BMI (Pre vs. Post, 41.15 ± 7.37 vs. 39.94 ± 6.86, *p* = 0.037) and changes in eating style (Pre vs. Post, 14.00 ± 10.40 vs. 9.82 ± 9.40, *p* = 0.017). BN and OSFED patients: no significant changes
11. Flaudias et al., 2020	Online survey	*N* = 5,738 undergraduate students (mean age = 21.2 ± 4.5, 38.3% at risk for ED symptoms)	France	March 2020 (10 days after lockdown)	SPS-10, HADS, PSS-10, EDI-2, SCOFF, IBSS.	Higher stress levels related to lockdown with a greater likelihood of reporting binge eating and dietary restrictions over the past 7 days (respectively: OR 5 1.12, 95% CI [1.04, 1.21], *p* < 0.004; OR 5 1.17, 95% CI [1.08, 1.26], *p* < 0.001) and over the course of the next 2 weeks (respectively: OR 5 1.33, 95% CI [1.19, 1.48], *p* < 0.001; OR 5 1.12, 95% CI [1.03, 1.21], *p* < 0.005). Greater COVID-19- media exposure associated with a higher likelihood of binge eating intentions over the course of the next 2 weeks (OR5 1.20, 95% CI [1.11, 1.31], *p* < 0.001).
12. Haddad et al., 2020	Online survey	*N* = 407 (mean age = 30.6 ± 10.1, 43.7% attending diet clinic for weight loss)	Lebanon	April 2020	EDE-Q, SBPS, LAS, BPAQ anger subscale	Physical exercise during confinement, female gender, BMI, and higher anxiety positively associated with higher severity of ED (EDE-Q subscales) both in dietician and in the general population groups. General population: lower physical contact with friends associated with shape (β = −0.86, *p* < 0.001) and weight (β = −0.72, *p* < 0.001) concerns. Dietician clients’ group: higher fear for COVID-19 associated with higher restraint (β = 0.06, *p* < 0.001), eating concern (β = 0.03, *p* = 0.002), shape concern (β = 0.07, *p* < 0.001) and weight concern (β = 0.05, *p* < 0.001) scores. Higher boredom and number of adults living in quarantine associated with shape (respectively β = 0.04, *p* < 0.001; β = 0.26, *p* = 0.002) and weight concerns (respectively, β = 0.03, *p* = 0.013; β = 0.27, *p* = 0.001). Restraint scores associated to lower boredom scores (β = −0.04, *p* = 0.001)
13. McCombie et al., 2020	Social media survey	*N* = 32 (mean age: 35.2 ± 10.3), 14 having a current ED and 18 an ED in the past	UK	May/June 2020	EDE-Q; DASS-21 Six open questions on pandemic experience	Evaluation of mechanisms contributing to ED exacerbation in 88% of cases. Positive aspects of life in lockdown were found in 72% of the sample. ED exacerbation was due to isolation (66%), worry/rumination, worsening of anxiety/depression (81%). Media impact was negative in 47%, disruption is structure and routine in 69%.
14. Phillipou et al., 2020	Online survey	*N* = 5.469 (mean age = 40.6 ± 13.7, 3.2% with self-report ED)	Australia	April 2020	DASS-21, EDE-Q	AN: increase in restricting behaviors (67.1%), and in binge eating (20.5%), in purging (18.2%), or in physical exercise (48.9%). Non-ED group: increase in food restrictions (27.6%), in binge eating (34.6%). Physical exercise significantly changed with an increase (34.8%) or a decrease (43.4%).
15. Puhl et al., 2020	Longitudinal cohort study	*N* = 584 secondary school students in 2010 (mean age = 24.6 ± 2.0)	USA, Minnesota	T0 = 2018 T1 = April 2020	EAT, C-EAT, QEWP-R, MFES, GSLTPAQ	Pre-pandemic experiences of weight stigma predicted higher levels of stress (β = 0.15, *p* = 0.001), depressive symptoms (β = 0.15, *p* < 0.001), eating to cope with stress (β = 0.16, *p* < 0.001), and increased likelihood of binge eating (OR = 2.88, *p* < 0.001) during pandemic.
16. Richardson et al., 2020	Retrospective study	*N* = 439 individuals with ED; *N* = 124 caregivers	Canada	March–April 2020	*Ad hoc*	The proportion of ED subjects who called NEDIC in 2020 describing dieting/restriction (*N* = 154 vs. *N* = 73 in 2019 vs. *N* = 47 in 2018, *p* < 0.001), over-exercising (*N* = 31 vs. *N* = 8 in 2019 vs. *N* = 6 in 2018, *p* = 0.008), perfectionism (*N* = 25 vs. *N* = 4 in 2019 vs. *N* = 1 in 2018, *p* < 0.001), purging (*N* = 61 vs. *N* = 25 in 2019 vs. *N* = 15 in 2018, p = 0.011), anxiety (*N* = 125 vs. *N* = 26 in 2019 vs. *N* = 27 in 2018, *p* < 0.001), and depression (*N* = 79 vs. *N* = 11 in 2019 vs. *N* = 12 in 2018, *p* < 0.001) significantly higher than both in 2018 and 2019. The number of teenagers was significantly higher in 2020 (28.70%; age 15–19 vs. 12.69% in 2018 and 11.22% in 2019) than in previous years.
17. Rolland et al., 2020	Online survey	*N* = 11.391	France	March–April 2020 (8–13 days after lockdown)	WEMWBS	Increase on evaluated addiction-related habits, including caloric/salty food (28.4%), screen use/abuse (64.6%), tobacco use (35.6%), cannabis use (31.2%), and alcohol use (24.8%). Factors of increase in caloric/salty food intake were female gender (OR 1.62, 95% CI 1.48–1.77), age <29 years (*p* < 0.001), having a partner (OR 1.19, 95% CI 1.06–1.35), being locked down in a more confined space (OR 1.02, 95% CI 1.01–1.03), being locked down alone (OR 1.29, 95% CI 1.11–1.49), and reporting current (OR 1.94, 95% CI 1.62–2.31) or past (OR 1.27, 95% CI 1.09–1.47) psychiatric treatment.
18. Scharmer et al., 2020	Online survey	*N* = 295 undergraduate participants (mean age = 19.7 ± 2.0)	USA, New York	March–April 2020	EDE-Q, FIVE, IUS, STAI-T, GLETQ, CET	Trait anxiety and the state of intolerance of uncertainty were both correlated with EDs (*R*^2^ = 0.46, *p* < 0.001, *R*^2^ = 0.30, *p* < 0.001). Compulsive exercise and EDs were influenced by intolerance of uncertainty as a trait (*R*^2^ = 0.21, *p* < 0.001, *R*^2^ = 0.35, *p* < 0.001). Subjects with lower levels of anxiety as a trait were more negatively affected by anxiety and uncertainty due to COVID-19, both on ED symptoms and in compulsive exercise.
19. Schlegl et al., 2020a	Online survey	*N* = 55 former females inpatients with BN (mean age = 24.4 ± 6.4)	Germany	May 2020	*Ad hoc*	Significant worsening of bulimic symptoms (49.1%), QoL (61.8%), depressive spectrum symptoms (75%), general ED psychopathology (80%). Binge eating increased (47.3%) as well as self-induced vomiting (364%), laxative use (9.1%), and diuretic abuse (7.3%). A high drive for activity was present in 61.8% of sample. More than 80% of patients with BN received face-to-face therapy before pandemic (81.8%) compared to 36.4% during pandemic, with only 20% opting for alternative treatment modalities, such as videoconference-based therapies.
20. Schlegl et al., 2020b	Online survey	*N* = 159 former inpatients with AN (22.4 ± 8.7)	Germany	May 2020	*Ad hoc*	Patients reported that eating, shape, and weight concerns, drive for physical activity, loneliness, sadness, and inner restlessness increased during pandemic (70%); 50% of patients reported no changes in restrictive eating, skipping meals, binge eating, and purging. Forty-one patients (25.8%) described positive consequences of pandemic, namely reduction in overall symptoms and enhancement of subjective responsibility to recover. Videoconference therapy was used in 26% and telephone contacts in 35% of samples.
21. Termorshuizen et al., 2020	Online survey	*N* = 511 from US (mean age 30.6 ± 9.4) *N* = 510 from NL, 92% currently have an ED	US, Netherland	April–May 2020	*Ad hoc*, GAD-7	Current ED: higher mean worry score (US, 4.59 ± 1.24 vs. 4.11 ± 1.22, *p* = 0.02; NL, 4.56 ± 1.17 vs. 4.05 ± 1.01, *p* = 0.02) and GAD-7 score (US, 12.87 ± 5.54 vs. 9.62 ± 5.36, *p* < 0.001; NL, 12.10 ± 5.37 vs. 8.43 ± 4.81, *p* < 0.001). Higher mean eating disorder impact in participants who reported difficulty accessing to treatment vs. those who received face-to-face or online treatment (2.69 ± 0.52 vs. 2.40 ± 0.58, *p* = 0.01)

*EES, emotional eating scale; PSS-10, perceived stress scale-10; GAD-7, generalized anxiety disorder-7; PHQ-9, patient health questionnaire-9; PSQI, Pittsburgh sleep quality index; GPAQ, global physical activity questionnaire; IPAQ-SF, international physical activity questionnaire short form; SWEMWBS, short Warwick-Edinburgh mental well-being scale; SMFQ, short mood and feelings questionnaire; SDBQL, short diet behaviors questionnaire for lockdowns; STBQL, short technology-use behaviors questionnaire for lockdowns; SSPQL, short social participation questionnaire for lockdowns; SDBQL, short diet behavior questionnaire for lockdowns; PSS-4, perceived stress scale-4; ESSI, ENRICHD social support instrument; SCI, Shapiro control inventory; RRS-ED, rumination response scale for eating disorders; CTQ-SF, childhood trauma questionnaire-short form; IES-R, impact of event scale-revised; ECR-R, experiences in close relationships-revised; EDE-Q, eating disorder examination-questionnaire; BSI, brief symptom inventory; SPS-10, 10-item social provision scale; HADS, hospital anxiety and depression scale; EDI-2, eating disorder inventory, 2nd edition; SCOFF, sick, control, one, fat, food; IBSS, ideal body stereotype scale; CIES, COVID isolation eating scale; SBPS, short boredom proneness scale; LAS, Lebanese anxiety scale; BPAQ, Buss–Perry scale; C-EAT, COVID-19 eating and activity over time; DASS-21, depression anxiety stress scales short version; M-FES, modified falls efficacy scale; QEWP-R, questionnaire on eating and weight patterns-R; EAT, eating and activity over time; FIVE, fear of illness evaluation; CET, compulsive exercise test; STAI-T, state-trait anxiety inventory-trait subscale; IUS, Intolerance of uncertainty scale-short form; GLETQ, Godin leisure-time exercise questionnaire; GSLTPAQ, Godin-Shephard leisure-time physical activity questionnaire; CAHSA, continuum of auditory hallucinations—state assessment; PSWQ, Penn state worry questionnaire; DASS-D, depression anxiety stress scales—depression subscale; SIAS, social interaction anxiety scale; SPS, social phobia scale; Y-BOCS, Yale–Brown obsessive compulsive scale—symptom checklist; SEES, Salzburg emotional eating scale; SCS, self-compassion scale: SCL-90-R, symptom checklist-90-revised; TCI-R, temperament and character inventory-revised; TFEQ-R18, the revised three factor nutrition questionnaire; YFAS-2, Yale food addiction scale 2.0*.

### Risk of Bias in Individual Studies

As already anticipated in Section Eligibility Criteria, the risk of bias of individual studies was not performed considering that no RCTs or systematic follow-up studies derived from RCTs were found.

### Data Synthesis

A meta-analysis could not be performed because of the lack of homogeneity among the resulting studies. In particular, it has been noted that studies varied in terms of how improvements were measured. Hence, this systematic review is summarized in a narrative synthesis.

## Results

**Figure 1:**
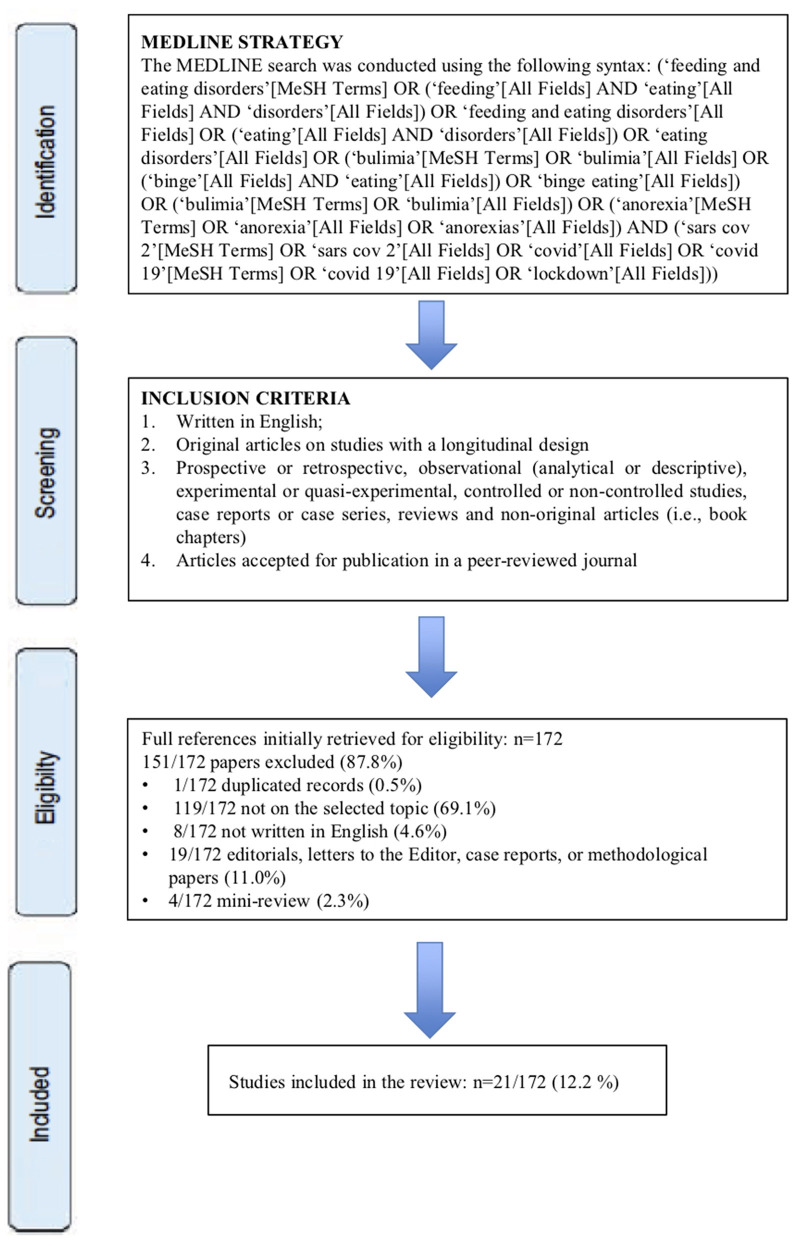
An overview of selection procedures.

After analyzing their titles and abstracts according to the eligibility criteria, 21 of 172 (12.2%) papers were included in the review, whereas 151 of 172 papers (87.8%) were excluded for the following reasons: 119 papers (69.1%) were not pertinent to the topic; 19 papers (11.0%) were excluded as these were editorials, letters to the editor, case reports, or methodological papers; 8 papers (4.6%) were excluded because they were not in English; 4 (2.3%) were mini-review, and finally there was 1 duplicated paper (0.5%). In the 21 selected studies, a total number of 29,108 subjects were enrolled (range: 10–11,391; mean/SD: 1,386 ± 2,800), 6,216 were men (21.4%), 22,703 were women (77.9%), and 189 (0.7%) were gender fluid or not declared. The mean age/SD of subjects was 30.2 ± 7.7. About 12 studies (57.1%) were online surveys; 4 (19.0%) were retrospective studies; 2 (9.5%) were qualitative studies; 2 (9.5%) were longitudinal cohort studies, and 1 was a social media survey (4.8%) (Figure 1).

The majority of the studies collected data through online platforms (an online survey). We subdivided the retrieved papers according to their main research areas as follows: studies on EDs in the general population; COVID-19 and lockdown consequences in patients with EDs; and key themes for patients with EDs during pandemic.

### Eating Spectrum in the General Population

During the COVID-19 pandemic, several studies investigated the prevalence of disturbed eating behaviors and related risk factors for exacerbations of EDs in the general population.

Al-Musharaf (2020) evaluated the impact of the COVID-19 pandemic on emotional eating (EE). Participants (*n* = 638) completed an online questionnaire including the Arabic version of the “*EE scale*” (EES) (Saade et al., 2019), the “*perceived stress scale*” (PSS) (Cohen et al., 1983), the “*generalized anxiety disorder-7*” (GAD-7) (Spitzer et al., 2006), the “*patient health questionnaire-9*” (PHQ-9) (Kroenke et al., 2001), the “*Pittsburgh sleep quality index*” (PSQI) (Buysse et al., 1989), and the “*global physical activity questionnaire*” (GPAQ) (World Health Organization., 2005). Moderate to high levels of EE were described by 52.8% of the sample. Moreover, 42% of the interviewed subjects reported depressive symptoms, together with high levels of anxiety (27.0%), and severe stress (12.5%). Highly perceived stress, body mass index (BMI), fat intake, the number of meals per day, sugary food consumption, and frequent fast food intake are positively correlated with the EE scores. The authors concluded that EE made an attempt to manage negative emotions during the stressful circumstances of the COVID-19 pandemic.

The study of Ammar et al. (2020) summarized preliminary data from an online survey on a sample of 1,047 participants from Asia, Africa, and Europe. The study was part of a larger survey, which was to assess the “effects of home confinement on multiple lifestyle behaviors during the COVID-19 outbreak (ECLB-COVID-19).” Questions were compared “*before*” vs. “*during*” confinement conditions. Changes in physical activity were assessed using the “*international physical activity questionnaire short form*” (IPAQ-SF) (Craig et al., 2003); dietary behaviors during lockdown were collected with a newly developed crisis-oriented questionnaire, the “*short diet behavior questionnaire for lockdowns*” (SDBQ-L) (Brach et al., 2020). Participants increased the consumption of unhealthy food, and the frequency of main meals, together with snacking between meals, with a subjective sense of “eating out of control.” The time spent for physical activity decreased under the confinement condition (days/week, 24%; min/day, 33.5%), whereas sedentary behavior (such as the daily sitting time) significantly increased (from 5 to 8 h/day).

One retrospective study investigated the effects of quarantine on BMI and sleep quality in Italian adults (*n* = 121) (Barrea et al., 2020). Data were collected at baseline, in an obesity outpatient clinic, and after 40 days of quarantine (T1), by telephone interviews, exploring physical activity (30 min/day of aerobic exercise YES/NO), working modalities (smart working YES/NO), and sleep quality (evaluated using the “*PSQI*”) (Buysse et al., 1989). All participants reported a reduction in physical activity during quarantine. After quarantine, a significant increase in BMI was observed, particularly in normal weight and obesity of grade I, and II subjects. Sleep quality was worsened except for severe obesity (grade III). Smart working negatively influenced sleep quality in all participants, with a greater worsening effect in men.

Elmacıoğlu et al. (2021) performed a retrospective cohort study on a sample of 1,036 volunteers, with the aim to assess the effects of the COVID-19 pandemic on nutritional behaviors and body weight changes of individuals. They found an increase in EE and uncontrolled eating behaviors as assessed with the “revised three factor nutrition questionnaire” (TFEQ-R18) (Karlsson et al., 2000), and found a change in BMI in approximately 35% of the sample.

Flaudias et al. (2020) utilized an online questionnaire administered to French undergraduate students (*n* = 5,738) 10 days after lockdown declaration. Participants were evaluated using the “*10-item social provision scale*” (SPS-10) (Cutrona and Russell, 1983), the “*hospital anxiety and depression scale*” (HADS) (Zigmond and Snaith, 1983), the “*PSS*” (Cohen et al., 1983), the body dissatisfaction and impulse regulation subscales of the “*ED inventory, 2nd edition*” (EDI-2) (Garner, 1991), the “*sick, control, one, fat, food*” (SCOFF) (Garcia et al., 2010), and the “*ideal body stereotype scale*” (IBSS) (Stice and Agras, 1998). Variables, such as lockdown-related stress, COVID-19-related stress, and media exposure to COVID-19, were also collected. The increased stress related to lockdown, the subjective suffering due to forced social distancing, and the daily routine disruptions were associated with a greater likelihood of reporting both binge eating and dietary restrictions. Moreover, the exposure to COVID-19 media news was associated with more severe binge eating behaviors. As expected, preexisting ED habits were the risk factors for the development of problematic eating behaviors during the COVID-19 pandemic.

Haddad et al. (2020) compared a group of people attending diet clinic for weight loss management (*n* = 177), and a group of subjects from the general population (*n* = 228) in Lebanon. People who were attending diet clinics should be the most affected due to disordered eating behaviors or weight and shape concerns, and the authors were interested in comparing the association of quarantine/confinement stressors and diet behaviors between the two groups of participants. The “*Lebanese anxiety scale*” (LAS) (Hallit et al., 2020), the “*short boredom proneness scale*” (SBPS) (Struk et al., 2017), the “*eating disorder examination-questionnaire*” (EDE-Q) (Fairburn and Beglin, 2008), and the anger subscale of the “*Buss–Perry scale*” (Buss and Perry, 1992) were administered. Approximately 44% of participants described a high fear of COVID-19. Physical exercise during confinement, female gender, BMI, and high anxiety levels were positively associated with higher severity of EDs, both in the clients of a dietician and in the general population. In the clients group of dieticians, the higher was the severity of EDs and the greater was the fear of COVID-19. This finding confirmed that stressful events might worsen altered eating behaviors, especially in vulnerable populations, such as the attendees of diet clinics. Social distancing was associated with shape and weight concerns only in the general population group.

A study by Phillipou et al. (2020) explored changes in eating and exercise behaviors following the official announcement of the COVID pandemic in Australia. They compared the subjects with and without a history of EDs in the general population sample aged >18 years. The main hypothesis of this study stated that subjects with a self-reported history of EDs might report significant changes in their habits more than the subjects without EDs, especially in four behaviors during pandemic, namely: restricting, binge eating, purging, and physical exercise. The study was conducted with a series of 72-h open anonymous online surveys each month and for 1 year, and was part of a larger survey, named as COLLATE (COVID-19 and you: mental health in Australia now survey). Of the original 8,014 individuals who were enrolled in the COLLATE survey, 5,469 completed the eating and exercise behavior section. Subjects with a positive history of EDs were 180, of whom 88 with AN, 23 with BN, 6 with BED, 4 with eating disorder not otherwise specified (ED-NOS), and 68 who did not specify the subtype of EDs. Given the diagnostic distribution, the most robust findings were from the AN subgroup. Indeed, these subjects noted a significant change in their eating and exercise behaviors, with an increase in restricting behaviors (67.1%), in binge eating (20.5%), or in purging (18.2%). Half of the subjects (48.9%) reported increased physical exercise to achieve more control on body shape and caloric intake. The same variables investigated in the subjects with no EDs did not reveal any changes in the amount of food restricted, or on binge eating or purging behaviors. Only the 27.6% of the subjects included in non-ED samples reported an increase in food restriction behaviors. Conversely, 34.6% reported an increase in binge eating spectrum behaviors. One-third of the sample (34.8%) showed an increase in the physical exercise routine, whereas 43.4% reported a decrease and the remaining reported no changes. To summarize, this study provided evidence regarding the worsening of eating spectrum signs, symptoms, and behaviors during pandemic, mainly when a history of EDs was present.

Weight stigma has been considered as a relevant variable in a study conducted on a subsample derived from a longitudinal cohort study, the EAT 2010–2018, and labeled as COVID-19 eating and activity over time study (C-EAT) (Puhl et al., 2020). The main aim of the C-EAT survey was to explore how weight-related health behaviors might change as a result of events related to COVID-19. To explain more in detail, it was considered how high body weight subjects perceived the social stigma linked to obesity during pandemic, and how such stigma contributed to the adoption of unhealthy eating behaviors and a lower physical activity. Individuals who experienced pre-pandemic weight stigma were compared with those who did not, in a sample of young people who were attending secondary school in Minneapolis, St. Paul (MN, USA), in 2009–2010. In agreement with the original assumption, majority of the overall sample reported that COVID-19 negatively affected mood and anxiety levels, raising stress, and exacerbating problems with food intake. The comparison between the two samples (stigma perceived before COVID vs. stigma perceived during COVID) revealed that pre-pandemic experiences of weight stigma predicted higher levels of stress, depressive symptoms, eating to cope with stress, and an increased likelihood of binge eating, during the COVID-19 pandemic. Furthermore, no significant interactions based on gender were found. In summary, young adults who experienced weight stigma before COVID showed increased vulnerability for binge eating and psychological distress during the pandemic. No information regarding other biopsychosocial variables such as the interpersonal environment of subjects or the role of attachment figures was provided.

Changes in caloric food intake were also found in a web-based survey, which was carried out in France during the 1st day of containment due to COVID-19, on more than 11,000 participants (Rolland et al., 2020). Apparently, respondents reported an increase in all evaluated addiction-related habits, including caloric/salty food (28.4%). However, the use/abuse of caloric foods was underrepresented if compared with screen use/abuse (64.6%), or tobacco (35.6%) and cannabis use (31.2%), and more represented than alcohol use (24.8%). Not surprisingly, female gender who were aged <29 years were locked down in a more confined space, were locked down alone, and reported to have current or past psychiatric treatment all were the factors of increased caloric/salty food intake.

Scharmer et al. (2020) investigated the potential relationships between the COVID-19 pandemic, anxiety levels, subjective intolerance of uncertainty, and the risk for ED pathology/compulsive exercise, in a sample of 295 undergraduate women in USA. Subjects were evaluated using the “*EDE-Q*” (Fairburn and Beglin, 2008; Berg et al., 2012), the “*fear of illness evaluation*” (FIVE) (Ehrenreich-May, 2020), the “*state-trait anxiety inventory-trait subscale*” (STAI-T) (Barnes et al., 2002), the “*compulsive exercise test*” (CET) (Taranis et al., 2011), the “*intolerance of uncertainty scale-short form*” (IUS) (Nicholas Carleton et al., 2007), and the “*Godin leisure-time exercise questionnaire*” (GLETQ) (Godin, 2011). They explored the effects of four different variables on EDs and compulsive exercise, namely the levels of anxiety correlated to COVID-19 (“*anxiety as a state*”), the intolerance to uncertainty correlated to COVID-19 (“*intolerance as a state*”), the “*anxiety levels*” as a stable trait, and the “*levels of intolerance to uncertainty*” as a trait. The results showed that both the state anxiety and the state of intolerance of uncertainty were positively related to EDs but not with compulsive exercise. By contrast, compulsive exercise and EDs were influenced by the intolerance of uncertainty as a trait. Moreover, subjects with lower levels of preexisting COVID-19 anxiety (i.e., anxiety as a trait) were the most negatively affected ones due to anxiety and uncertainty caused by COVID-19, both on ED symptoms and in compulsive exercise. In summary, the authors concluded that stable traits of anxiety and uncertainty were more relevant in worsening EDs and compulsive exercise than the current levels of the same subjective variables.

### COVID-19 and Lockdown Consequences in Patients With EDs

An online survey study has been performed on a sample of 74 patients with EDs who have been already followed-up before the COVID-19 outbreak, and re-evaluated during lockdown with the aim to assess psychological and psychopathological factors that might impact on ED symptoms (Baenas et al., 2020). Patients were administered with the ED inventory-2 (Garner, 1991), the yale addiction scale-2 (Gearhardt et al., 2016), the symptom checklist-90 revised (SCL-90-R) (Derogatis, 1994), and the temperament and character inventory revised (TCI-R) (Cloninger et al., 1994). Moreover, patients were interviewed with a semi-structured brief telephone survey on employment status, confinement compliance, affected environment, and the presence of the company during the lockdown. The results of this study were controversial. Thus, the authors concluded that a significant proportion of patients experienced the worsening of ED symptoms, even if this finding was only in 19 patients (25.7%). Majority of the samples (*n* = 55) did not report a significant variation of their symptoms. However, performing a path analysis, they found a direct effect of the TCI-R dimension “self-directedness” on the worsening of EDs. A limitation of this study was that they compared a total of 32 different dimensions or domains from EDI-2, SCL-90R, YFAS, and TCI-R on a sample of 74 patients and that they did not find significant differences comparing patients who were worsened (*n* = 19) and patients who were not (*n* = 55), except for the abovementioned self-directedness.

Branley-Bell and Talbot (2020) collected the data from 80 subjects with current EDs and 49 in recovery with the aim to understand the impact of the COVID-19 pandemic in people with experiences of ED. A mixed-method online survey was developed. Participants completed open-ended questions related to the impact of lockdown and some validated questionnaires to assess mental well-being [*short Warwick-Edinburgh mental well-being scale* (SWEMWBS)] (Tennant et al., 2007), *perceived stress* scale (PSS) (Cohen et al., 1983), social support [*ENRICHD social support instrument* (ESSI)] (Vaglio et al., 2004), perceived control [*Shapiro control inventory* (SCI)] (Shapiro, 1994), and rumination [*rumination response scale for EDs* (RRS-ED)] (Cowdrey and Park, 2011). Majority of participants (86.7%) reported that ED symptoms worsened as a result of pandemic. Only two participants reported an improvement in symptoms. As expected, participants with EDs in remission exhibited higher mental well-being, lower perceived stress, higher social support, and perceived control when compared to participants with current ED.

The study of Fernández-Aranda et al. (2020) aimed at evaluating the impact of home confinement, in terms of changes in eating behaviors and symptoms, in a sample of 87 patients with EDs and 34 patients with obesity. Patients with EDs were recruited in centers representative of the public and private sectors of ED treatment services in Barcelona: 55 were suffering from AN, 18 from BN, and 14 from other specified feeding or eating disorders (OSFED), according to diagnostic and statistical manual of mental disorders, fifth edition (DSM-5) criteria. Patients with obesity were recruited at the endocrinology unit of the Clinic Hospital of Barcelona. The authors collected data using the COVID isolation eating scale (CIES), a newly created questionnaire to investigate four different domains: circumstances during the confinement, effects of the confinement on ED symptoms, behavioral, and psychopathological impact of the confinement, and the evaluation of online intervention, considering “before” vs. “after the confinement.” The psychometric properties of the CIES were analyzed, and a factor analysis confirmed the rational structure of the CIES.

In contrast with previous studies, the disordered eating improved in almost all participants during confinement. The impact of COVID-19 pandemic was mixed and varied in the given diagnostic distribution. Surprisingly, patients with AN reported a decrease in ED symptoms and in emotion dysregulation; patients with obesity showed a significant decrease in BMI and in eating symptomatology; and patients with BN and OSFED exhibited no significant changes before and after the confinement. Patients with OSFED deserved special attention: although the changes between pre- and post-confinement did not reach the statistical significance, they reported the highest impairment in psychopathology. This finding highlighted the need to promptly identify the vulnerable subjects that might be more sensitive to adverse events. Concerning the use of telemedicine, patients with AN expressed the greatest dissatisfactions and difficulties with online treatments.

The longitudinal study of Castellini et al. (2020) tested three hypotheses: (1) patients with EDs might represent a more vulnerable population to the effects of the COVID-19 pandemic; (2) a recovery process might be affected by the lockdown circumstances; and (3) the factors preceding pandemic might be associated with the worsening of psychopathology during lockdown. Data from 74 patients were collected using the current DSM-5 diagnosis of AN or BN, attending an individual enhanced cognitive behavioral therapy (Fairburn, 2008) at an outpatient clinic for patients with EDs. A group of 97 healthy controls (HCs) were also enrolled. Both groups were evaluated before lockdown (T1) and during lockdown (T2), while patients with AN and BN were also evaluated at the beginning of the treatment (T0). T0, T1, and T2 assessment included the “*brief symptom inventory*” (BSI) (Derogatis, 1994), and the “*EDE-Q*” (Fairburn and Beglin, 2008). At baseline, the “*childhood trauma questionnaire-short form*” (CTQ-SF) (Bernstein et al., 2003) and the “*experiences in close relationships-revised*,” (ECR-R) (Fraley et al., 2000) were also administered. Moreover, during lockdown (T2), the “*impact of event scale-revised*” (IES-R) (Weiss and Marmar, 1997), adapted for the COVID-19 pandemic, was administered. Not surprisingly, patients with EDs were more vulnerable to lockdown effects than those with HC, as confirmed by their increase in pathological eating behaviors (objective binge eating and compensatory physical exercise). Moreover, the ED group reported higher COVID-19-related post-traumatic stress (PTS) symptoms in comparison with the HC group. While comparing baseline to T1, patients with EDs reported an improvement of general psychopathology and ED-specific symptoms, objective binge eating and compensatory physical exercise. Moreover, BMI increased significantly in patients with AN while remaining stable in patients with BN. Comparisons between pre- and post-lockdown (T1–T2) revealed that patients with EDs increased physical exercise during lockdown despite the initial improvement. Some specific differences between diagnostic subgroups emerged: patients with AN reported an improvement in BMI and specific ED psychopathology, whereas patients with BN exhibited an increase of objective binge eating. Despite 10 full remissions and 19 partial remissions at T1, 10 subjects reported a relapse into BN at T2. No significant change was found in the HC group. Interestingly, the main factor of increase in pathological physical exercise was household arguments, while the fear of safety for the loved ones predicted a higher increase in binge eating episodes. Childhood trauma and insecure attachment style resulted in the factors for COVID-19-related PTS symptoms in patients with AN. In conclusion, patients with EDs, and particularly those suffering from BN, were more vulnerable to the impact of lockdown. Indeed, lockdown interfered with a recovery process in terms of relapses into pathological eating behaviors, such as compulsive physical exercise and exacerbations of binge eating. The results seemed to be more controversial in patients with AN as BMI and ED psychopathology constantly improved despite the exacerbation of compulsive exercise.

Changes in help-seeking behaviors from subjects with EDs and their caregivers due to the COVID pandemic were the focus of a retrospective Canadian study that analyzed part of the information collected by the National Eating Information Center (CEDIC). CEDIC was a national non-profit organization that, since 1985, operated toll-free helpline and an instant chat service for patients with EDs (Richardson et al., 2020). The proportion of patients with EDs who called the CEDIC describing dieting/restriction, over-exercising, perfectionism, purging, anxiety, and depression was significantly higher in 2020 than in 2018 and 2019. The most relevant issues in 2020 were related to the difficulties in accessing treatments because of the pandemic, and to an urgent need for support, due to the worsening of symptoms. Subjective feelings of losing control over eating symptoms and behaviors and with significant changes in food intake were also described. Interestingly, there was a significantly higher number of teenagers who contacted CEDIC in 2020 compared to 2018 and 2019 (28.70%; age 15–19 vs. 12.69% in 2018 and 11.22% in 2019), maybe because teenagers were not attending school in person, and had no regular access to their usual support networks and structures (guidance counselors, or teachers).

Schlegl et al. (2020a,b) published two papers based on an online survey for patients with EDs who were interviewed during the COVID-19 pandemic. The first study was carried out in a sample of 55 former female inpatients with BN (age range 17–46 years) (Schlegl et al., 2020a). They were evaluated by a self-developed questionnaire assessing psychological consequences of pandemic, such as the overall impact on ED symptoms and on the quality of life (QoL), the adverse effect on therapies, the incidence of new symptoms, worries regarding infections, relapses, food insecurity or job, general psychopathology, interpersonal conflicts, and healthcare utilization. Enjoyable activities, virtual social contact with friends, and mild physical exercises were also considered to be among the helpful strategies adopted by patients to face pandemic stressful events. In summary, the results were discouraging: more than half of the interviewed patients described a significant worsening of their bulimic symptoms and QoL. They also described the worsening of depressive spectrum signs and symptoms together with general psychopathology levels in around 80% of the cases. Binge eating increased as well as self-induced vomiting, laxative use, and diuretic abuse. A high drive for activity was present in 60% of the sample. Moreover, patients described difficulties with daily routines, especially meals. The worsening of symptoms was also related to the interruption of face-to-face therapies in more than half of patients, with only 20% opting for alternative treatment modalities such as videoconference-based therapies.

The second study was conducted on a sample of 159 former patients with AN (age range 14–62 years), with the same modalities as mentioned in a previous study (Schlegl et al., 2020b). The results were less discouraging. Indeed, even if 70% of patients reported that eating, shape, and weight concerns, driving for physical activity, loneliness, sadness, and inner restlessness increased during pandemic, there was also a subsample of 41 patients (25.8%) describing positive consequences in terms of a reduction in overall symptoms and of an enhancement of subjective responsibility to recover. Moreover, videoconference therapy was used by 26% and telephone contacts was used by 35% of patients.

A two-site online study in USA and the Netherlands recruited subjects with EDs (AN, BN, and BED) *via* social media, or through emails when they were already enrolled in previous surveys (Termorshuizen et al., 2020). Variables, such as the exposure to the virus, diagnosis in self or relatives, impact of COVID-19 on the health or employment of family members, and level of lockdown, were collected. The impact of the pandemic on EDs was evaluated using a four-point Likert-scale by asking general questions on health subjective perception and specific questions on symptoms, such as binge eating. A seven-point Likert scale was also administered to explore worries about exposure and/or contracting the virus. Finally, a free text item inquired about changes to EDs treatments, including the frequency of treatment sessions and subjective perception of quality of treatments. Patients with AN described mainly fears about the availability of foods chosen for their “special” meals. Moreover, they reported an exacerbation of food restriction. Patients with BN and BED increased binge and purge episodes. Positive effects of the pandemic were described also in this survey on both the interpersonal (greater connection with relatives) and the motivational side. All groups described difficulties in accessing their treatments.

An online survey was conducted in UK on a very small sample of 32 subjects >17 years old with current or previous EDs, enrolled with posts on Twitter (McCombie et al., 2020). Participants were asked to describe their ED status (current/partial recovery/full recovery), and to report on their illness duration. They were evaluated with the “*EDE-Q*,” the “*depression, anxiety, and stress scales-version 21*” (DASS-21), and with a six open-ended questions interview, exploring the subjective experience of pandemic. Eating disorder symptoms were described as exacerbated by pandemic, and by the associated changes in lifestyle in 88% of the cases. Physical and psychological isolation, worry about the future, fear around a “*return to normal life*,” and changes in daily routine were the main topics. Unexpectedly, 72% of participants described a sort of “*positive effect of having more space and time for healing and self-care*,” and having less pressure to engage in social activities.

### Key Themes for Patients With EDs During Pandemic

Three qualitative studies explored the impact of the COVID-19 outbreak and associated lockdown measures on patients with self-reported EDs (Branley-Bell and Talbot, 2020; Brown et al., 2020) or patients with AN and their caregivers (Clark Bryan et al., 2020).

As a consequence of the intense media reports on COVID-19 (the so-called “*infodemic*”), the levels of anxiety increased, with an exacerbation of the fear of contamination and washing compulsions (Clark Bryan et al., 2020). Some patients revealed that the fear of contamination concerned primarily the health of the elderly and more vulnerable the loved ones rather than themselves (Brown et al., 2020).

The introduction of lockdown measures and social isolation was associated with negative mental health outcomes, sense of loneliness, the tendency to become more focused on food, rumination over food intake, disordered eating behaviors, such as restrictions or binge eating, and compulsive physical exercise (Branley-Bell and Talbot, 2020; Brown et al., 2020; Clark Bryan et al., 2020). Moreover, the imposed functional restrictions disrupted the established daily routines that are needed to be adapted to the uncertainty of a continuously changing environment. Again, patients reported increased anxiety for the need to deconstruct rigid regimens and to adjust them to the current situation (Brown et al., 2020) and experienced reduced motivation for recovery (Branley-Bell and Talbot, 2020; Clark Bryan et al., 2020). This led to the exacerbation of ED symptoms and to the engagement in disordered eating behaviors (Branley-Bell and Talbot, 2020).

Enhanced exposure to triggering contents, such as messages promoting in-house physical exercise or food recipes, and an easy access to extreme content (such as, pro-AN content) was detrimental for most participants (Branley-Bell and Talbot, 2020). Only social support perceived through social media (WhatsApp, etc.) represented a source for reducing the lockdown sense of isolation (Branley-Bell and Talbot, 2020).

Brown et al. (2020) pointed out that lockdown measures might also have a positive effect and lead to recovery, depending on the working and living situation of participants (alone vs. with a family or a partner or friends), and ED progression. Some participants reported increased self-responsibility, intentionality in planning their actions, and increased attempts for a self-management that improved their EDs behaviors.

These studies highlighted the role of the accessibility to professional support as a crucial determinant of mental well-being: patients complained about discrepancies in service provision, premature discharge, and suspension of treatments that increased anxiety, and feelings of being abandoned (Branley-Bell and Talbot, 2020; Brown et al., 2020; Clark Bryan et al., 2020). Although the continuation of treatments through online delivery was described as a positive factor, this was not seen as a “valuable alternative” but just as the “only possibility” (Branley-Bell and Talbot, 2020).

## Discussion

The study we selected regarding the effects of the pandemic on eating habits was heterogeneous for sample selection, study design, and outcome measures, leading to inhomogeneous results. However, we found five relevant issues as summarized below.

### The Role of Changes in Daily Routines

Patients with EDs experienced during pandemic the worsening of their preoccupation with weight and body shape, due to the difficulties in continuing their compensatory behaviors, including compulsive physical exercise (Castellini et al., 2020; Clark Bryan et al., 2020; Phillipou et al., 2020; Richardson et al., 2020; Schlegl et al., 2020a,b). Disruption in physical activities and routines was relevant also in general population samples or in clinical samples from endocrinology units (Ammar et al., 2020; Barrea et al., 2020; Haddad et al., 2020; Scharmer et al., 2020). As postulated in the “*social zeitgeber*” hypothesis, unstable, or disrupted daily routines might lead to circadian rhythm instability and, in vulnerable individuals, to mood instability, sleep disorders, and dietary disruptions (Frank et al., 2005, 2008). According to this model, psychosocial factors would interact with chronobiological mechanisms to create and/or facilitate a pathway toward psychopathology.

### Restrictions or Difficulties in Healthcare Access

This factor was relevant in the three studies (Brown et al., 2020; Clark Bryan et al., 2020; Termorshuizen et al., 2020). patients with EDs might have unstable physical conditions requiring frequent medical monitoring and lab tests, but in several countries the usual medical services have been converted to the services exclusively dedicated to patients with COVID, thus limiting the access for other medical reasons (Marazziti et al., 2020). Telemedicine check-ups have been proposed as an alternative approach, but monitoring weight changes or vital signs was limited in a long-distance follow-up. Moreover, videoconference-based therapies were adopted by a limited number of subjects as described by Schlegl et al. (2020a).

### The Role of Stress and Anxiety

Several studies before pandemic highlighted how patients with EDs might show higher levels of intolerance for uncertainty compared to the general population (Brown et al., 2017). Moreover, routine changes leading to body weight modifications may exacerbate additional concerns in patients with EDs. It is well-known that ED-stereotyped behaviors play a role in controlling anxiety levels and in managing negative emotions (Kesby et al., 2017). We found that uncertainty and anxiety due to pandemic contributed to an increase in subjective discomfort and raised the risk of compulsory eating as a strategy to cope with stress (Flaudias et al., 2020; Puhl et al., 2020). Changes in emotion regulation had also an impact on EE (Al-Musharaf, 2020; Elmacıoğlu et al., 2021). In one study (Baenas et al., 2020), TCI-R self-directedness was found as the only discriminating factor between patients with EDs who did and did not experience the worsening of their symptoms. However, this study was focused on the evaluation of a small sample (*n* = 74) using 32 different domain/dimensions, which belongs to four different scales.

### Social Isolation and Home Confinement

The type of home confinement (with family members, in small places, alone, or with a partner, etc.) modified both subjective well-being and addiction-related habits, including caloric/salty food (Rolland et al., 2020). Moreover, as already noticed, home confinement had negative effects on physical activity (Ammar et al., 2020). Patients with EDs experienced the worsening of their symptoms as a direct consequence of deprivation in relationships with the significant ones, especially when an insecure attachment style was present (Castellini et al., 2020). Only two studies found a “positive” effect of confinement on eating-related styles. Fernández-Aranda et al. (2020) studied on a sample of patients with EDs and subjects with obesity, with several limitations, such as the retrospective design, the wide age range (from 13 to 77 years), and the gender distribution (104 women vs. 17 men). Brown et al. (2020) studied only on 15 subjects who consented to take part in a Skype interview. A third study (McCombie et al., 2020) found non-univocal results, but was conducted on a very small sample (*n* = 32) recruited with posts on Twitter. One study (Baenas et al., 2020) apparently found a worsening in patients with EDs who are already followed-up before the COVID-19 outbreak and evaluated by phone during a lockdown. The study sample was small (*n* = 74), and only 19 patients (25.7%) experienced a clinically significant worsening of their symptoms, whereas the majority of the sample (*n* = 55) did not, raising questions on the validity of such long-distance assessments. Interestingly, this study utilized a path analysis that revealed a direct effect of the TCI-R dimension of self-directedness on the deterioration of ED symptoms during lockdown.

### The Problem of EDs in Adolescents and Young Adults

Before pandemic, ~85% of adolescents were unhappy with their body (Ricciardelli and McCabe, 2001), and 50–60% reported “*disturbed eating behaviors*” (e.g., fasting, taking diet pills or laxatives, vomiting, and binge eating) (Croll et al., 2002; Quick and Byrd-Bredbenner, 2013). Maladaptive eating behaviors in adolescents were associated with depressive symptoms and low self-esteem, before the pandemic (Stice and Whitenton, 2002; Neumark-Sztainer et al., 2006). We were wondering on the “*specific*” role of COVID-19 and on the relationships between new potential stressors and the worsening/triggering of EDs in adolescents/young adults, considering that there is a lack of evidence on this topic. Thus, the mean age of the studies we found ranged from 19 (Scharmer et al., 2020) to 54 years (Clark Bryan et al., 2020). The study by Puhl et al. (2020) was on secondary school students, enrolled in a 10-year longitudinal cohort study, with a mean age of 24 years in pandemic (Puhl et al., 2020). Only one study reported that the number of teenagers who referred to an anonymous helpline chat for psychological distress (NEDIC) was significantly higher in 2020 than in the previous 2 years (Richardson et al., 2020).

## Study limitations

Across studies, the main weaknesses were due to the limited assessment of pre-lockdown ED symptoms and to the absence of comparison groups. Moreover, in some studies, the ED diagnoses were self-reported, and the specific information on disorders was not collected. Participants were mostly recruited online, *via* social media, resulting in samples being biased toward those who were familiar with the use of technology. However, this choice was unavoidable during the lockdown. The discrepancy in data collection points (the early stage vs. late stage of lockdown) made it difficult to compare the main findings. No studies accounted for attrition rates. Follow-up data were absent for the majority of studies. There were online surveys or retrospective studies (see, for example, Ammar et al., 2020; Fernández-Aranda et al., 2020) in which the variables were compared between pre- vs. post-pandemic. There are only two longitudinal cohort studies (Castellini et al., 2020; Puhl et al., 2020) that compared pre- vs. post-pandemic behaviors or tried to find predictors of negative outcomes during pandemic. As summarized previously, we found the studies that varied in terms of study design, population sample, and with no structured intervention proposed. Finally, 50 different outcome measures or psychological questionnaires were used, raising questions on how to compare findings from such a number of scales. As a consequence, the results of this review must be interpreted with caution due to the abovementioned limitations.

## Data Availability Statement

The original contributions presented in the study are included in the article/supplementary material, further inquiries can be directed to the corresponding author/s.

## Author Contributions

All authors gave their substantial contributions to conception and design, data acquisition, data analysis, and interpretation. All authors gave contributions in drafting the article or critically revising it for important intellectual content, and gave their final approval of the version to be published. All authors agree to be accountable for all aspects of the work in ensuring that questions related to the accuracy or integrity of the work are appropriately investigated and resolved.

## Conflict of Interest

The authors declare that the research was conducted in the absence of any commercial or financial relationships that could be construed as a potential conflict of interest.

## Publisher's Note

All claims expressed in this article are solely those of the authors and do not necessarily represent those of their affiliated organizations, or those of the publisher, the editors and the reviewers. Any product that may be evaluated in this article, or claim that may be made by its manufacturer, is not guaranteed or endorsed by the publisher.

## References

[B1] Al-Musharaf S. (2020). Prevalence awend predictors of emotional eating among healthy young saudi women during the COVID-19 pandemic. Nutrients 12, 1–17. 10.3390/nu12102923 32987773 PMC7598723

[B2] Ammar A. Brach M. Trabelsi K. Chtourou H. Boukhris O. Masmoudi L. . (2020). Effects of COVID-19 home confinement on eating behaviour and physical activity: results of the ECLB-COVID19 international online survey. Nutrients 12, 1583. 10.3390/nu12061583 32481594 PMC7352706

[B3] Baenas I. Caravaca-Sanz E. Granero R. Sánchez I. Riesco N. Testa G. . (2020). COVID-19 and eating disorders during confinement: analysis of factors associated with resilience and aggravation of symptoms. Eur. Eat. Disord. Rev. 28, 855–863. 10.1002/erv.2771 32815293 PMC7461472

[B4] Barnes L. L. B. Harp D. Jung W. S. (2002). Reliability generalization of scores on the Spielberger state-trait anxiety inventory. Educ. Psychol. Meas. 62, 603–618. 10.1177/0013164402062004005

[B5] Barney A. Buckelew S. Mesheriakova V. Raymond-Flesch M. (2020). The COVID-19 pandemic and rapid implementation of adolescent and young adult telemedicine: challenges and opportunities for innovation. J. Adolesc. Health 67, 164–171. 10.1016/j.jadohealth.2020.05.006 32410810 PMC7221366

[B6] Barrea L. Pugliese G. Framondi L. Di Matteo R. Laudisio D. Savastano S. . (2020). Does SARS-Cov-2 threaten our dreams? Effect of quarantine on sleep quality and body mass index. J. Transl. Med. 18, 318. 10.1186/s12967-020-02465-y 32811530 PMC7432549

[B7] Berg K. C. Peterson C. B. Frazier P. Crow S. J. (2012). Psychometric evaluation of the eating disorder examination and eating disorder examination-questionnaire: a systematic review of the literature. Int. J. Eat. Disord. 45, 428–438. 10.1002/eat.20931 21744375 PMC3668855

[B8] Bernstein D. P. Stein J. A. Newcomb M. D. Walker E. Pogge D. Ahluvalia T. . (2003). Development and validation of a brief screening version of the Childhood Trauma Questionnaire. Child Abuse Neglect 27, 169–190. 10.1016/S0145-2134(02)00541-0 12615092

[B9] Brach M. Trabelsi K. Chtourou H. Boukhris O. Masmoudi L. Bouaziz B. . (2020). Effects of COVID-19 home confinement on physical activity and eating behaviour preliminary results of the ECLB-COVID19 international online-survey. MedRxiv 2020.05.04.20072447. 10.1101/2020.05.04.20072447 PMC7352706 32481594

[B10] Branley-Bell D. Talbot C. V. (2020). Exploring the impact of the COVID-19 pandemic and UK lockdown on individuals with experience of eating disorders. J. Eat. Disord. 8, 1–12. 10.1186/s40337-020-00319-y 32874585 PMC7444862

[B11] Brown M. Robinson L. Campione G. C. Wuensch K. Hildebrandt T. Micali N. (2017). Intolerance of uncertainty in eating disorders: a systematic review and meta-analysis. Eur. Eat. Disord. Rev. 25, 329–343. 10.1002/erv.2523 28544668

[B12] Brown S. M. Opitz M. C. Peebles A. I. Sharpe H. Duffy F. Newman E. (2020). A qualitative exploration of the impact of COVID-19 on individuals with eating disorders in the UK. Appetite 156, 104977. 10.1016/j.appet.2020.104977 32991945 PMC7521890

[B13] Buss A. H. Perry M. (1992). The aggression questionnaire. J. Pers. Soc. Psychol. 63, 452–459. 10.1037//0022-3514.63.3.452 1403624

[B14] Buysse D. J. Reynolds C. F. Monk T. H. Berman S. R. Kupfer D. J. (1989). The Pittsburgh sleep quality index: a new instrument for psychiatric practice and research. Psychiatry Res. 28, 193–213. 10.1016/0165-1781(89)90047-4 2748771

[B15] Castellini G. Cassioli E. Rossi E. Innocenti M. Gironi V. Sanfilippo G. . (2020). The impact of COVID-19 epidemic on eating disorders: a longitudinal observation of pre versus post psychopathological features in a sample of patients with eating disorders and a group of healthy controls. Int. J. Eat. Disord. 53, 1855–1862. 10.1002/eat.23368 32856333 PMC7461528

[B16] Clark Bryan D. Macdonald P. Ambwani S. Cardi V. Rowlands K. Willmott D. . (2020). Exploring the ways in which COVID-19 and lockdown has affected the lives of adult patients with anorexia nervosa and their carers. Eur. Eat. Disord. Rev. 28, 826–835. 10.1002/erv.2762 32643844 PMC7362064

[B17] Cloninger R. Przybeck T. Svrakic D. Wetzel R. (1994). TCI-The Temperament and Character Inventory (TCI): A Guide to its Development and Use. Available online at: http://www.researchgate.net/publication/264329741

[B18] Cohen S. Kamarck T. Mermelstein R. (1983). A global measure of perceived stress. J. Health Soc. Behav. 24, 385–396. 10.2307/2136404 6668417

[B19] Conversano C. Marchi L. Miniati M. (2020). Psychological distress among healthcare professionals involved in the COVID-19 emergency: vulnerability and resilience factors. Clin. Neuropsychiatry 17, 94–96. 10.36131/CN20200212 PMC8629057 34908976

[B20] Cowdrey F. A. Park R. J. (2011). Assessing rumination in eating disorders: principal component analysis of a minimally modified ruminative response scale. Eat. Behav. 12, 321–324. 10.1016/j.eatbeh.2011.08.001 22051368

[B21] Craig C. L. Marshall A. L. Sjöström M. Bauman A. E. Booth M. L. Ainsworth B. E. . (2003). International physical activity questionnaire: 12-country reliability and validity. Med. Sci. Sports Exerc. 35, 1381–1395. 10.1249/01.MSS.0000078924.61453.FB 12900694

[B22] Croll J. Neumark-Sztainer D. Story M. Ireland M. (2002). Prevalence and risk and protective factors related to disordered eating behaviors among adolescents: relationship to gender and ethnicity. J. Adolesc. Health 31, 166–175. 10.1016/S1054-139X(02)00368-3 12127387

[B23] Cutrona C. Russell D. W. (1983). The provisions of social relationships and adaptation to stress in Advances in Personal Relationships, eds Jones W. H. Perlman D. (Greenwich, CT: JAI Press), 37–67.

[B24] Derogatis L. R. (1994). SCL-90-R: Administration, Scoring and Procedures Manual, 3rd Edn. Minneapolis, MN: National Computer Systems, Inc. 10.1017/S0033291700048017

[B25] Ehrenreich-May J. (2020). Fear of Illness and Virus Evaluation Scale - Adult Form. Available online at: Available online at: https://adaa.org/sites/default/files/UofMiamiFear%20of%20Illness%20and%20Virus%20Evaluation%20_(FIVE)%20scales%20for%20Child-%2C%20Parent-%20and%20Adult-Report.pdf

[B26] Elmacıoğlu F. Emiroğlu E. Ülker M. T. Özyılmaz Kırcali B. Oruç S. (2021). Evaluation of nutritional behaviour related to COVID-19. Public Health Nutr. 24, 512–518. 10.1017/S1368980020004140 33070798 PMC7737137

[B27] Fairburn C. G. (2008). Cognitive Behavior Therapy and Eating Disorders. New York, NY: Guilford Press.

[B28] Fairburn C. G. Beglin S. (2008). Eating Disorder Examination Questionnaire (EDE-Q). New York: Guilford Press.

[B29] Fernández-Aranda F. Munguía L. Mestre-Bach G. Steward T. Etxandi M. Baenas I. . (2020). COVID Isolation Eating Scale (CIES): analysis of the impact of confinement in eating disorders and obesity—a collaborative international study. Eur. Eat. Disord. Rev. 28, 871–883. 10.1002/erv.2784 32954595 PMC7537123

[B30] Flaudias V. Iceta S. Zerhouni O. Rodgers R. F. Billieux J. Llorca P.-M. . (2020). COVID-19 pandemic lockdown and problematic eating behaviors in a student population. J. Behav. Addict. 9, 826–835. 10.1556/2006.2020.00053 32976112 PMC8943668

[B31] Fraley R. Waller N. Brennan K. (2000). An item response theory analysis of self-report measures of adult attachment. J. Pers. Soc. Psychol. 78, 350–365. 10.1037//0022-3514.78.2.350 10707340

[B32] Frank E. Kupfer D. J. Thase M. E. Mallinger A. G. Swartz H. A. Fagiolini A. M. . (2005). Two-year outcomes for interpersonal and social rhythm therapy in individuals with bipolar I disorder. Arch. Gen. Psychiatry 62, 996–1004. 10.1001/archpsyc.62.9.996 16143731

[B33] Frank E. Soreca I. Swartz H. A. Fagiolini A. M. Mallinger A. G. Thase M. E. . (2008). The role of interpersonal and social rhythm therapy in improving occupational functioning in patients with bipolar I disorder. Am. J. Psychiatry 165, 1559–1565. 10.1176/appi.ajp.2008.07121953 18829872 PMC3308335

[B34] Garcia F. D. Grigioni S. Chelali S. Meyrignac G. Thibaut F. Dechelotte P. (2010). Validation of the French version of SCOFF questionnaire for screening of eating disorders among adults. World J. Biol. Psychiatry 11, 888–893. 10.3109/15622975.2010.483251 20509759

[B35] Garner D. (1991). Eating Disorder Inventory-2: Professional Manual. Odessa, FL: Psychological Assessment Resources.

[B36] Gearhardt A. N. Corbin W. R. Brownell K. D. (2016). Development of the yale food addiction scale version 2.0. Psychol. Addict. Behav. 30, 113–121. 10.1037/adb0000136 26866783

[B37] Godin G. (2011). The Godin-Shephard leisure-time physical activity questionnaire. Health Fitness J. Canada 4, 18–22. 10.14288/hfjc.v4i1.8225799030

[B38] Haddad C. Zakhour M. Bou Kheir M. Haddad R. Al Hachach M. Sacre H. . (2020). Association between eating behavior and quarantine/confinement stressors during the coronavirus disease 2019 outbreak. J. Eat. Disord. 8, 40. 10.1186/s40337-020-00317-0 32879730 PMC7458649

[B39] Hallit S. Obeid S. Haddad C. Hallit R. Akel M. Haddad G. . (2020). Construction of the Lebanese Anxiety Scale (LAS-10): a new scale to assess anxiety in adult patients. Int. J. Psychiatry Clin. Pract. 24, 270–277. 10.1080/13651501.2020.1744662 32228282

[B40] Hensley. (2020). Why the Coronavirus Pandemic is Triggering Those With Eating Disorders – National. Globalnews.ca. Available online at: https://globalnews.ca/news/6735525/eating-disorder-coronavirus/ (accessed January 8, 2021).

[B41] Karlsson J. Persson L. O. Sjostrom L. Sullivan M. (2000). Psychometric properties and factor structure of the Three-Factor Eating Questionnaire (TFEQ) in obese men and women. Results from the Swedish Obese Subjects (SOS) study. Int. J. Obes. Relat. Metab. Disord. 24, 1715–1725. 10.1038/sj.ijo.0801442 11126230

[B42] Kesby A. Maguire S. Brownlow R. Grisham J. R. (2017). Intolerance of uncertainty in eating disorders: an update on the field. Clin. Psychol. Rev. 56, 94–105. 10.1016/j.cpr.2017.07.002 28710918

[B43] Kroenke K. Spitzer R. L. Williams J. B. W. (2001). The PHQ-9: validity of a brief depression severity measure. J. Gen. Intern. Med. 16, 606–613. 10.1046/j.1525-1497.2001.016009606.x 11556941 PMC1495268

[B44] Marazziti D. Pozza A. Di Giuseppe M. Conversano C. (2020). The psychosocial impact of COVID-19 pandemic in Italy: a lesson for mental health prevention in the first severely hit European country. Psychol. Trauma Theory Res. Pract. Policy 12, 531–533. 10.1037/tra0000687 32525387

[B45] McCombie C. Austin A. Dalton B. Lawrence V. Schmidt U. (2020). Now it’s just old habits and misery”–understanding the impact of the covid-19 pandemic on people with current or life-time eating disorders: a qualitative study. Front. Psychiatry 11, 589225. 10.3389/fpsyt.2020.589225 33192736 PMC7653176

[B46] McMenemy. (2020). Coronavirus and Eating Disorders: “I Feel Selfish Buying Food” - BBC News. Available online at: https://www.bbc.com/news/uk-england-51962964 (accessed: January 8, 2021).

[B47] Miniati M. Marazziti D. (2019). Psychopharmacological options for adult patients with anorexia nervosa: the patients’ and carers’ perspectives integrated by the spectrum model. CNS Spectrums 24, 225–226. 10.1017/S1092852917000700 28990542

[B48] Moher D. Liberati A. Tetzlaff J. Altman D. G. (2009). Preferred reporting items for systematic reviews and meta-analyses: the PRISMA statement. PLoS Med. 6:e1000097. 10.1371/journal.pmed.1000097 19621072 PMC2707599

[B49] Neumark-Sztainer D. Wall M. Guo J. Story M. Haines J. Eisenberg M. (2006). Obesity, disordered eating, and eating disorders in a longitudinal study of adolescents: how do dieters fare 5 years later? J. Amer. Diet. Assoc. 106, 559–568. 10.1016/j.jada.2006.01.003 16567152

[B50] Nicholas Carleton R. Sharpe D. Asmundson G. J. G. (2007). Anxiety sensitivity and intolerance of uncertainty: requisites of the fundamental fears? Behav. Res. Ther. 45, 2307–2316. 10.1016/j.brat.2007.04.006 17537402

[B51] Orrù G. Marzetti F. Conversano C. Vagheggini G. Miccoli M. Ciacchini R. . (2021). Secondary traumatic stress and burnout in healthcare workers during COVID-19 outbreak. Int. J. Environ. Res. Publ. Health 18, 337. 10.3390/ijerph18010337 33466346 PMC7794988

[B52] Phillipou A. Meyer D. Neill E. Tan E. J. Toh W. L. Van Rheenen T. E. . (2020). Eating and exercise behaviors in eating disorders and the general population during the COVID-19 pandemic in Australia: initial results from the COLLATE project. Int. J. Eat. Disord. 53, 1158–1165. 10.1002/eat.23317 32476163 PMC7300745

[B53] Poli A. Gemignani A. Conversano C. (2020). The psychological impact of Sars-Cov-2 quarantine: OBSERVATIONS through the lens of the polyvagal theory. Clin. Neuropsychiatry 17, 112–114. 10.36131/CN20200216 PMC8629065 34908980

[B54] Puhl R. M. Lessard L. M. Larson N. Eisenberg M. E. Neumark-Stzainer D. (2020). Weight stigma as a predictor of distress and maladaptive eating behaviors during COVID-19: longitudinal findings from the EAT study. Ann. Behav. Med. Publ. Soc. Behav. Med. 54, 738–746. 10.1093/abm/kaaa077 32909031 PMC7499477

[B55] Quick V. M. Byrd-Bredbenner C. (2013). Disturbed eating behaviours and associated psychographic characteristics of college students. J. Hum. Nutr. Diet. 26, 53–63. 10.1111/jhn.12060 23627697

[B56] Ricciardelli L. A. McCabe M. P. (2001). Children’s body image concerns and eating disturbance: a review of the literature. Clin. Psychol. Rev. 21, 325–344. 10.1016/S0272-7358(99)00051-3 11288604

[B57] Richardson C. Patton M. Phillips S. Paslakis G. (2020). The impact of the COVID-19 pandemic on help-seeking behaviors in individuals suffering from eating disorders and their caregivers. Gen. Hosp. Psychiatry 67, 136–140. 10.1016/j.genhosppsych.2020.10.006 33129138 PMC10277602

[B58] Rolland B. Haesebaert F. Zante E. Benyamina A. Haesebaert J. Franck N. (2020). Global changes and factors of increase in caloric/salty food intake, screen use, and substance use during the early COVID-19 containment phase in the general population in France: survey study. JMIR Publ. Health Surveill. 6, e19630. 10.2196/19630 32589149 PMC7505683

[B59] Saade S. Hallit S. Haddad C. Hallit R. Akel M. Honein K. . (2019). Factors associated with restrained eating and validation of the Arabic version of the restrained eating scale among an adult representative sample of the Lebanese population: a cross-sectional study. J. Eat. Disord. 7, 24. 10.1186/s40337-019-0254-2 31346465 PMC6636024

[B60] Schardt C. Adams M. B. Owens T. Keitz S. Fontelo P. (2007). Utilization of the PICO framework to improve searching PubMed for clinical questions. BMC Med. Inform. Decis. Mak. 7, 16. 10.1186/1472-6947-7-16 17573961 PMC1904193

[B61] Scharmer C. Martinez K. Gorrell S. Reilly E. E. Donahue J. M. Anderson D. A. (2020). Eating disorder pathology and compulsive exercise during the COVID-19 public health emergency: examining risk associated with COVID-19 anxiety and intolerance of uncertainty. Int. J. Eat. Disord. 53, 2049–2054. 10.1002/eat.23395 33098579 PMC8817895

[B62] Schlegl S. Maier J. Meule A. Voderholzer U. (2020a). Eating disorders in times of the COVID-19 pandemic—Results from an online survey of patients with anorexia nervosa. Int. J. Eat. Disord. 53, 1791–1800. 10.1002/eat.23374 32841413 PMC7461418

[B63] Schlegl S. Meule A. Favreau M. Voderholzer U. (2020b). Bulimia nervosa in times of the COVID-19 pandemic—results from an online survey of former inpatients. Eur. Eat. Disord. Rev. 28, 847–854. 10.1002/erv.2773 32767858 PMC7436773

[B64] Shapiro D. H. (1994). Shapiro Control Inventory (SCI) Manual | Control Research. Available online at: http://controlresearch.net/shapiro-control-inventory-manual.html

[B65] Spitzer R. L. Kroenke K. Williams J. B. W. Löwe B. (2006). A brief measure for assessing generalized anxiety disorder: The GAD-7. Arch. Int. Med. 166, 1092–1097. 10.1001/archinte.166.10.1092 16717171

[B66] Stice E. Agras W. S. (1998). Predicting onset and cessation of bulimic behaviors during adolescence: a longitudinal grouping analysis. Behav. Ther. 29, 257–276. 10.1016/S0005-7894(98)80006-3

[B67] Stice E. Whitenton K. (2002). Risk factors for body dissatisfaction in adolescent girls: a longitudinal investigation. Dev. Psychol. 38, 669–678. 10.1037/0012-1649.38.5.669 12220046

[B68] Struk A. A. Carriere J. S. A. Cheyne J. A. Danckert J. (2017). A short boredom proneness scale: development and psychometric properties. Assessment 24, 346–359. 10.1177/1073191115609996 26467085

[B69] Taranis L. Touyz S. Meyer C. (2011). Disordered eating and exercise: development and preliminary validation of the compulsive exercise test (CET). Eur. Eat. Disord. Rev. 19, 256–268. 10.1002/erv.1108 21584918

[B70] Tennant R. Hiller L. Fishwick R. Platt S. Joseph S. Weich S. . (2007). The Warwick-Dinburgh mental well-being scale (WEMWBS): Development and UK validation. Health Qual. Life Outcomes 5, 63. 10.1186/1477-7525-5-63 18042300 PMC2222612

[B71] Termorshuizen J. D. Watson H. J. Thornton L. M. Borg S. Flatt R. E. Macdermod C. M. . (2020). Early impact of covid-19 on individuals with eating disorders: a survey of ~1000 individuals in the United States and the Netherlands. MedRxiv 2020.05.28.20116301. 10.1101/2020.05.28.20116301 32720399

[B72] Touyz S. Lacey H. Hay P. (2020). Eating disorders in the time of COVID-19. J. Eat. Disord. 8, 19. 10.1186/s40337-020-00295-3 32337045 PMC7170399

[B73] Vaglio J. Conard M. Poston W. S. O’Keefe J. Haddock C. K. House J. . (2004). Testing the performance of the ENRICHD social support instrument in cardiac patients. Health Qual. Life Outcomes 2, 24. 10.1186/1477-7525-2-24 15142277 PMC434528

[B74] Weiss D. S. Marmar C. R. (1997). The impact of event scale-revised in Assessing Psychological Trauma and PTSD: A Practitioner’s Handbook, eds Wilson J. P. Keane T. M. (New York: Guilford Press), 399–411.

[B75] Weissman R. S. Bauer S. Thomas J. J. (2020). Access to evidence-based care for eating disorders during the COVID-19 crisis. Int. J. Eat. Disord. 53, 369–376. 10.1002/eat.23279 32338400 PMC7267278

[B76] World Health Organization. (2005). Global Physical Activity Questionnaire Analysis Guide GPAQ Analysis Guide Global Physical Activity Questionnaire (GPAQ) Analysis Guide. Available online at: http://www.who.int/chp/steps/GPAQ/en/index.html

[B77] Zigmond A. S. Snaith R. P. (1983). The hospital anxiety and depression scale. Acta Psychiatr. Scand. 67, 361–370. 10.1111/j.1600-0447.1983.tb09716.x 6880820

